# Vertical photon sorting by stacking silicon and germanium nanopillars for broadband absorbers

**DOI:** 10.1515/nanoph-2023-0014

**Published:** 2023-03-27

**Authors:** Rongyang Xu, Takumi Morimoto, Junichi Takahara

**Affiliations:** Graduate School of Engineering, Osaka University, 2-1 Yamadaoka, Suita, Osaka 565-0871, Japan; Photonics Center, Graduate School of Engineering, Osaka University, 2-1 Yamadaoka, Suita, Osaka 565-0871, Japan

**Keywords:** degenerate critical coupling, metasurface, stacked perfect absorbers

## Abstract

Perfect absorbers based on all-dielectric metasurfaces exhibit great potential in photodetection, photovoltaics, and imaging applications. This study proposes and demonstrates an all-dielectric broadband absorber comprising subwavelength-thick nanopillar Mie resonators in the visible light range. This nanopillar functions as a perfect absorber based on degenerate critical coupling with a characteristic “degenerate critical length.” At this length, the nanopillars are capable of achieving perfect absorption. Beyond this length, the peak of perfect absorption is not affected with further increases in the length of the nanopillars. Hence, this study realizes broadband absorption via the stacking of amorphous silicon and germanium nanopillars with the same width at different peak absorption wavelengths. The absorption spectra are almost independent of the order of the stacked structures; hence, the stacked nanopillars in the specific stacking order can behave as a vertical photon sorter, sorting photons based on the wavelength. This study provides a systematic route to the realization of broadband absorbers with vertical photon sorting capability via the vertical stacking of nanopillars.

## 1 Introduction

Perfect absorbers, which absorb most of the incident light, have great potential for applications in photodetection [1–3], imaging [4, 5], sensing [6, 7], thermal emission [8], and photovoltaics [9]. They can be realized using metasurfaces comprising subwavelength-thick artificial nanostructure arrays [10–13]. The optical properties of metasurfaces can be tuned through changes to the geometry and the period of the nanostructures. Plasmonic metasurfaces typically use a tri-layer metal-dielectric-metal (MDM) structure to achieve perfect absorption [8, 14]. However, metals are lossy in the visible and near-infrared (NIR) ranges. Consequently, a large fraction of the incident light is attenuated by the metal of the MDM perfect absorbers and converted into heat, thereby limiting the application of MDM perfect absorbers in photodetection, photovoltaics, and imaging.

Metasurfaces composed of high-refractive-index dielectric materials can also achieve perfect absorption. Dielectric metasurfaces support Mie-type resonant modes such as electric dipole (ED), magnetic dipole (MD), electric quadrupole (EQ), and magnetic quadrupole (MQ) modes [15–18]. For a freestanding subwavelength-thick film comprising dielectric nanostructures, the maximum absorption is limited to 50% provided only one resonant mode is excited [19–21]. Hence, dielectric Mie resonators are typically combined with metal plates to achieve perfect absorption [22]. Perfect absorption of monolayer Mie resonators can be achieved based on degenerate critical coupling (DCC). In 2014, DCC-based perfect absorption was first proposed and achieved using photonic crystals [20, 21]. In 2017, the concept of DCC-based perfect absorption was applied to dielectric Mie resonators in the terahertz range [23]. Subsequently, DCC-based perfect absorbers have been proposed for the visible [24, 25] and NIR ranges [26–28]. However, these DCC-based perfect absorbers can achieve only narrowband perfect absorption because they require the radiative loss *γ* of the resonant modes to be equal to the material loss *δ*. Although high-order and non-radiative modes with low *γ* can be used to achieve narrowband perfect absorption [25, 28], the use of DCC-based perfect absorbers in photovoltaic and thermophotovoltaic applications is hindered. Moreover, because of the use of single-size periodic Mie resonators, these perfect absorbers cannot distinguish between different wavelengths of incident light in imaging applications [5].

Because the MDM structure exhibits strong field confinement, the combination of multi-size resonators in a unit cell [11, 29] can result in MDM perfect absorbers achieving broadband perfect absorption and sorting of photons of different wavelengths. In contrast, dielectric Mie resonators typically exhibit weak field confinement, which can be greatly improved using DCC-based nanopillar perfect absorbers [30]. The DCC-based perfect absorption results in rapid attenuation of the incident light, which does not propagate in the nanopillar Mie resonators. Hence, nanopillar absorbers composed of different dielectric materials can be stacked to achieve broadband absorption and photon sorting.

This study proposes a nanopillar perfect absorber composed of amorphous silicon (a-Si) based on the DCC. Owing to the strong field confinement, the nanopillars composed of a-Si and amorphous germanium (a-Ge) is stacked to achieve broadband absorbers. In addition, the a-Si nanopillar is placed on top of the a-Ge nanopillar, which results in the stacked perfect absorber functioning as a photon sorter capable of selectively absorbing incident light of various wavelengths.

## 2 Results and discussion

### 2.1 Degenerate critical coupling

Nanopillar perfect absorbers are achieved based on the DCC [30]. The DCC can be divided into degenerate and critical coupling [20, 21, 23]. For a mirror-symmetric system (e.g., a dielectric Mie resonator in air), an incident beam from one port can be considered as a combination of two pairs of counter-propagating beams with half the amplitude of the incident electric field. The pair of beams without a phase difference is regarded as an even eigenexcitation, which can be coupled to an even mode (e.g., ED mode). The other pair of beams with a phase difference is an odd eigenexcitation, which can be coupled to an odd mode (e.g., MD mode). The even and odd modes have different symmetries; therefore, in case of a moderate incident electric field and linear system response, the two modes are orthogonal and uncoupled [20, 23]. The total absorption *A* is a linear sum of the absorption of the even and odd modes (*A* = *A*_even_ + *A*_odd_). To achieve more than 50% absorption, the even and odd modes should degenerate in wavelength, that is, the peak wavelengths of both modes should be the same. If *γ* of the two modes is equal to *δ*, these modes are critically coupled, with each mode absorbing 50% of the incident light. Perfect absorption can be achieved by satisfying the conditions of degenerate and critical coupling. The absorption equation based on coupled-mode theory is as follows [23]:

(1) $$\begin{array}{c} A=\frac{2\gamma_{1}\delta_{1}}{{\left(\omega -\omega_{1}\right)}^{2}+{\left(\gamma_{1}+\delta_{1}\right)}^{2}}+\frac{2\gamma_{2}\delta_{2}}{{\left(\omega -\omega_{2}\right)}^{2}+{\left(\gamma_{2}+\delta_{2}\right)}^{2}}, \end{array}$$

where *ω*, *ω*_1_, and *ω*_2_ are the resonant frequencies of the incident light and the even and odd modes, respectively, and *γ*_1_, *δ*_1_, *γ*_2_, and *δ*_2_ are the radiative and material losses of the even and odd modes, respectively. Thus, perfect absorption at resonance can be achieved by satisfying the conditions of degenerate (*ω*_1_ = *ω*_2_) and critical coupling (*γ*_1_ = *δ*_1_ and *γ*_2_ = *δ*_2_).

### 2.2 Silicon nanopillar and perfect absorber

This study employs nanopillar Mie resonators to achieve a wideband perfect absorption. The ED mode of nanopillar Mie resonators exhibits a larger *γ* than that of nanodisk Mie resonators [24]. This is because a compactly arranged nanodisk Mie resonator array supports a toroidal electric dipole (TED) mode, which has a radiation pattern similar to that of the ED mode [31, 32]. The destructive interference between the ED and TED modes leads to a lower *γ* ED mode [31–35]. Hence, perfect absorbers composed of nanodisk Mie resonators achieve narrowband perfect absorption [24, 26].

**Figure 1:**
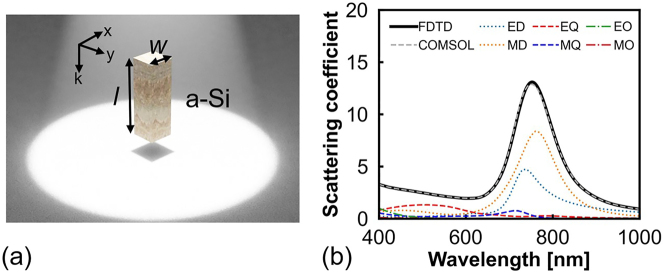
Study of a single a-Si nanopillar. (a) Schematic of an a-Si nanopillar Mie resonator. (b) Scattering coefficient of the nanopillar Mie resonator with *w* = 120 nm and *l* = 380 nm. The scattering coefficient is defined as the ratio of the scattering cross-section to the geometric cross-section of the nanopillar Mie resonator.

Figure 1a shows a schematic of an a-Si nanopillar Mie resonator with a width *w* and length *l* in air. First, a 3D electromagnetic simulator (ANSYS Lumerical FDTD) based on the finite-difference time-domain (FDTD) method is used to calculate the total scattering cross-section. Subsequently, multipole decomposition is performed using COMSOL (COMSOL Multiphysics) to study the multipole resonant modes of the nanopillar Mie resonator. The complex refractive index of a-Si used in the simulations is obtained from Pierce’s study [36]. As shown in Figure 1b, the total scattering coefficient of the FDTD simulation (black solid line) is consistent with that of COMSOL (grey dashed line). Further, the ED and MD modes with similar peak wavelengths are dominant at approximately 750 nm. Higher-order modes of weaker responses, such as EQ, MQ, electric octupole (EO), and magnetic octupole (MO) modes, are not considered in this study.

**Figure 2:**
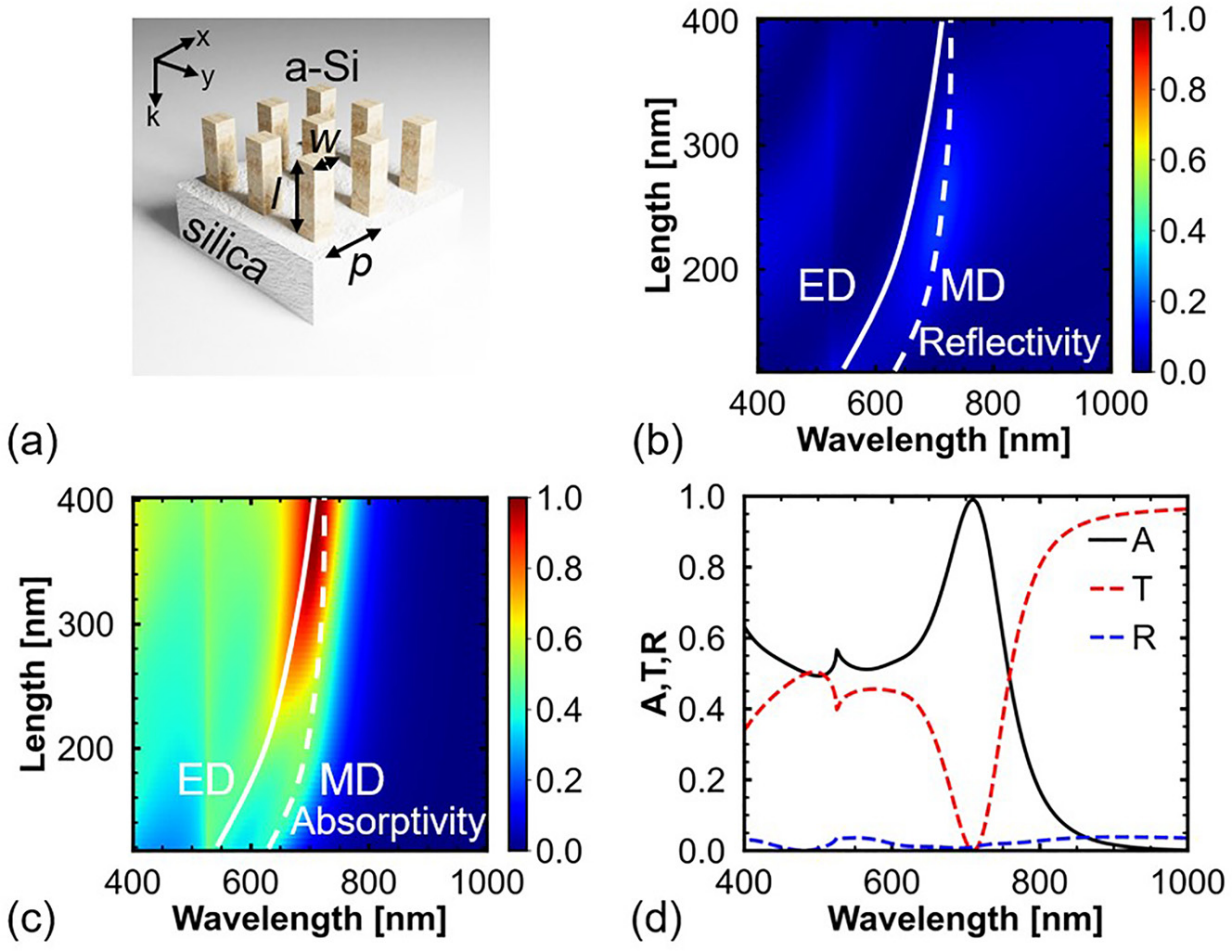
Study of a-Si nanopillar arrays. (a) Schematic of a nanopillar Mie resonator array. (b) Reflection and (c) absorption spectral maps of the Mie resonators with *w* = 120 nm, *l* = 120–400 nm, and *p* = 360 nm. (d) Absorption, transmission, and reflection spectra of the Mie resonators with *l* = 380 nm.

Subsequently, the collective response of a nanopillar Mie resonator array is studied. Figure 2a shows the schematic of the model used in the simulation (Supporting Information S1 presents the simulation setup). The nanopillar Mie resonators are placed on a silica substrate at period *p*. Owing to the same width of the nanopillars in the *x*- and *y*-directions, the polarization of the incident light does not affect the response of the proposed structures in this study. Figure 2b shows the reflection spectral map of the Mie resonator arrays with *l* = 120–400 nm. The reflection of the Mie resonator arrays is very low, regardless of the value of *l*. This is because, in the absence of an excited resonant mode, the small filling factor of the nanopillar Mie resonators results in an effective medium with a refractive index of approximately 1.3, which is slightly greater than that of air [37]. The small difference in the refractive index between the effective medium and air results in the low reflection. The trends for the ED and MD modes are indicated by white solid and dashed lines, respectively. Although these two modes are not degenerate in wavelength, the reflectivity of the peaks of the ED and MD modes is still low. For example, at *l* = 180 nm, the reflectivity of the MD mode at 677 nm is as low as 12%. This is because the reflection peaks of the ED and MD modes arise from destructive interference between the incident and scattered fields in the forward direction [26, 38, 39]. The high absorption of the MD mode results in a reduction in the scattered field. Hence, the destructive interference between the incident and scattered fields in the forward direction is suppressed, and the reflectivity decreases.

Figure 2c shows the absorption spectral map of the Mie resonator arrays. At *l* = 120 nm, the peaks of the ED and MD modes are separated. Further, the peak of the MD mode is not affected by the Rayleigh anomaly, and the absorptivity of the MD mode is approximately 40%. In addition, because of the high extinction coefficient of a-Si (*γ* < *δ*), the MD mode at 620 nm is not critically coupled (*γ* = *δ*). With increases in *l*, the peak wavelength of the ED mode exhibits a larger redshift than that of the MD mode; therefore, the peaks of these two modes approach each other, and the total absorptivity increases. Finally, at *l* = 380 nm, the peak wavelengths of the two modes degenerate, and perfect absorption is achieved at 709 nm, as shown in Figure 2d. Owing to low reflection, the unabsorbed light continues to propagate forward.

**Figure 3:**
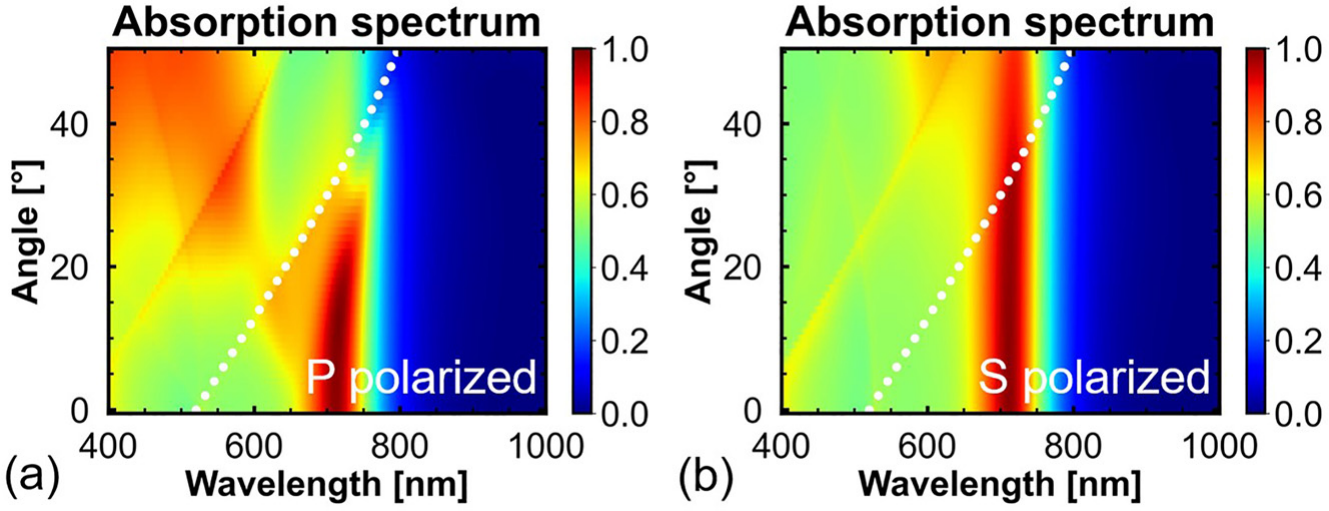
Angle-dependent absorption spectral maps of the a-Si perfect absorber with *w* = 120 nm, *l* = 380 nm, and *p* = 360 nm under (a) *p*-polarized and (b) *s*-polarized incident light. The white dotted lines indicate the wavelength of the Rayleigh anomaly.

Figure 3 shows the angle-dependent absorption spectral maps of the a-Si perfect absorber. As shown in Figure 3a, for p-polarized incident light, the absorption of the a-Si perfect absorber exceeds 80% at an incident angle of 26°. However, the peak of perfect absorption is affected by the Rayleigh anomaly, the wavelength of which is indicated by white dotted lines [40]. Lattice resonance occurs if the wavelength of the Rayleigh anomaly is close to the peak wavelength of the Mie resonant modes [41]. In this case, the conditions of the DCC-based perfect absorption are not satisfied; therefore, the absorption decreases. As shown in Figure 3b, for *s*-polarized incident light, the absorption peak is insensitive to changes in the angle of the incident light. The absorptivity is still higher than 86% at an incident angle of 50°. Hence, the absorption spectrum of the proposed a-Si perfect absorber is insensitive to the change in the incident angle.

**Figure 4:**
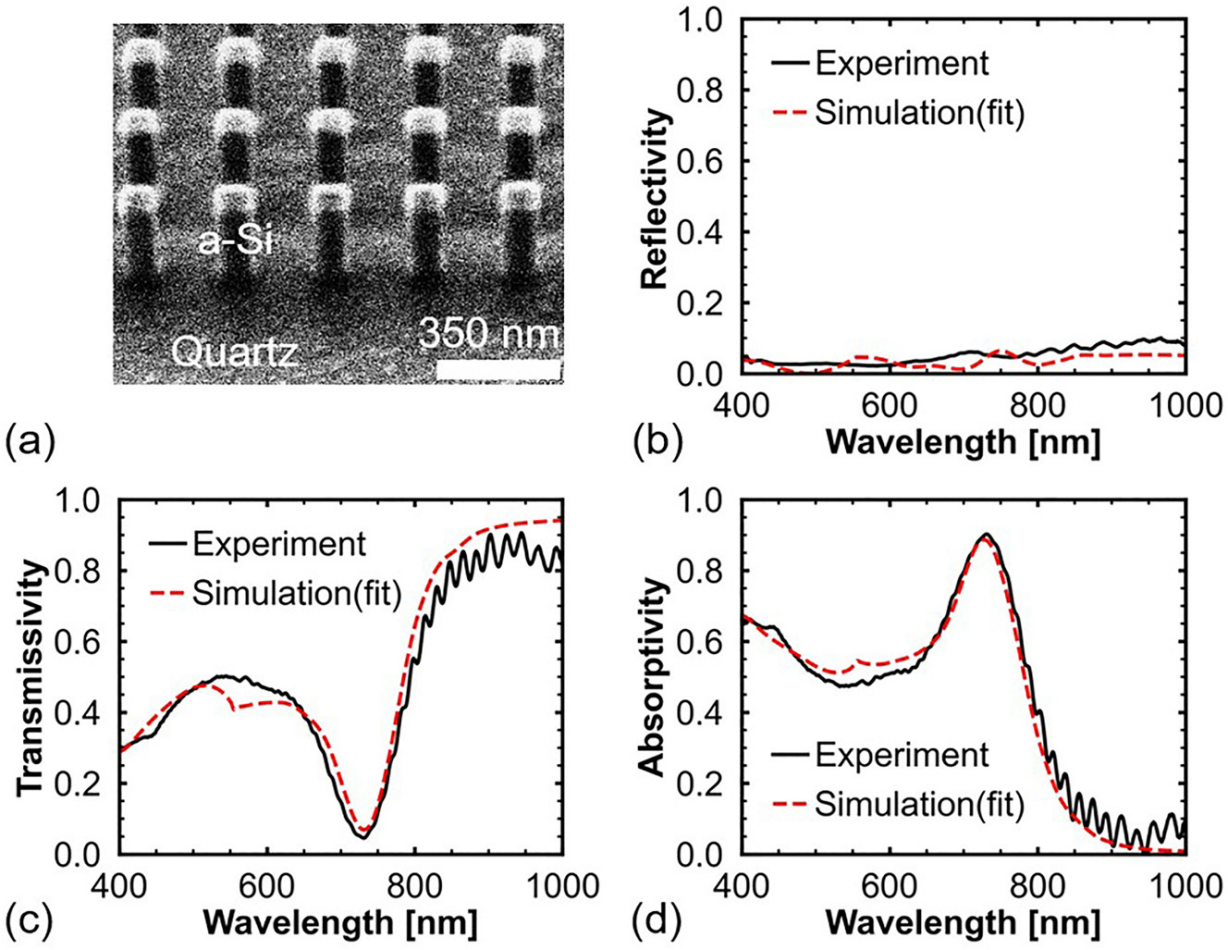
Experimental results of the proposed a-Si nanopillars. (a) Focused ion beam image of the fabricated a-Si nanopillar Mie resonators taken at an angle of 40°. (b) Measured reflection and (c) transmission spectra of the fabricated sample. (d) Absorption spectrum calculated by 1 − *T* − *R*.

A sample was fabricated using a silicon-on-quartz substrate to demonstrate the proposed a-Si perfect absorber (Supporting Information S2 presents the fabrication process). The refractive index of quartz is similar to that of silica. Hence, changing the substrate from silica to quartz has a negligible effect on the final results. Figure 4a shows an image of the fabricated sample captured at an angle of 40° using a focused ion beam system. Figure 4b–d shows the measured reflection (*R*), transmission (*T*), and calculated absorption (*A*) spectra of the sample, respectively (Supporting Information S3 presents the measurement equipment). In addition, numerical simulations were performed to study the measured spectra, and the simulated spectra are plotted with red dashed lines in Figure 4. The simulated spectra are consistent with the measured and calculated spectra. Moreover, the reflection of the Mie resonator array is very low, and unabsorbed light passed through the quartz substrate. The sample exhibits 90% absorption at 730 nm. Although the angle of the incident light in the measurement is 0–8.6°, such a small incident angle has a negligible effect on the absorption spectrum. The absorption rate being slightly below 99% can be attributed to two reasons. First, the geometry of the fabricated nanopillar Mie resonators is not identical to the model used in the simulation. The upper-end width of the nanopillars is wider than the width of 120 nm used in the simulation because of a slightly longer dose time used in the electron beam lithography process. In addition, the sidewalls of the fabricated nanopillars are tilted owing to lateral etching in the process of dry etching. The difference in geometry leads to a redshift of the absorption peak (Supporting Information S4 provides detailed information on fabrication errors and the effect of changing the upper-end width of nanopillars on the absorption spectrum). Second, *k* of a-Si is wavelength-dependent, and the wavelength of the absorption peak of the fabricated sample is longer than that of the simulation results in Figure 2d. The *k* value of a-Si at 730 nm is slightly smaller (*γ* > *δ*) than the required *k* to achieve the critical coupling of the ED and MD modes.

### 2.3 Stacked nanopillars and broadband absorbers

**Figure 5:**
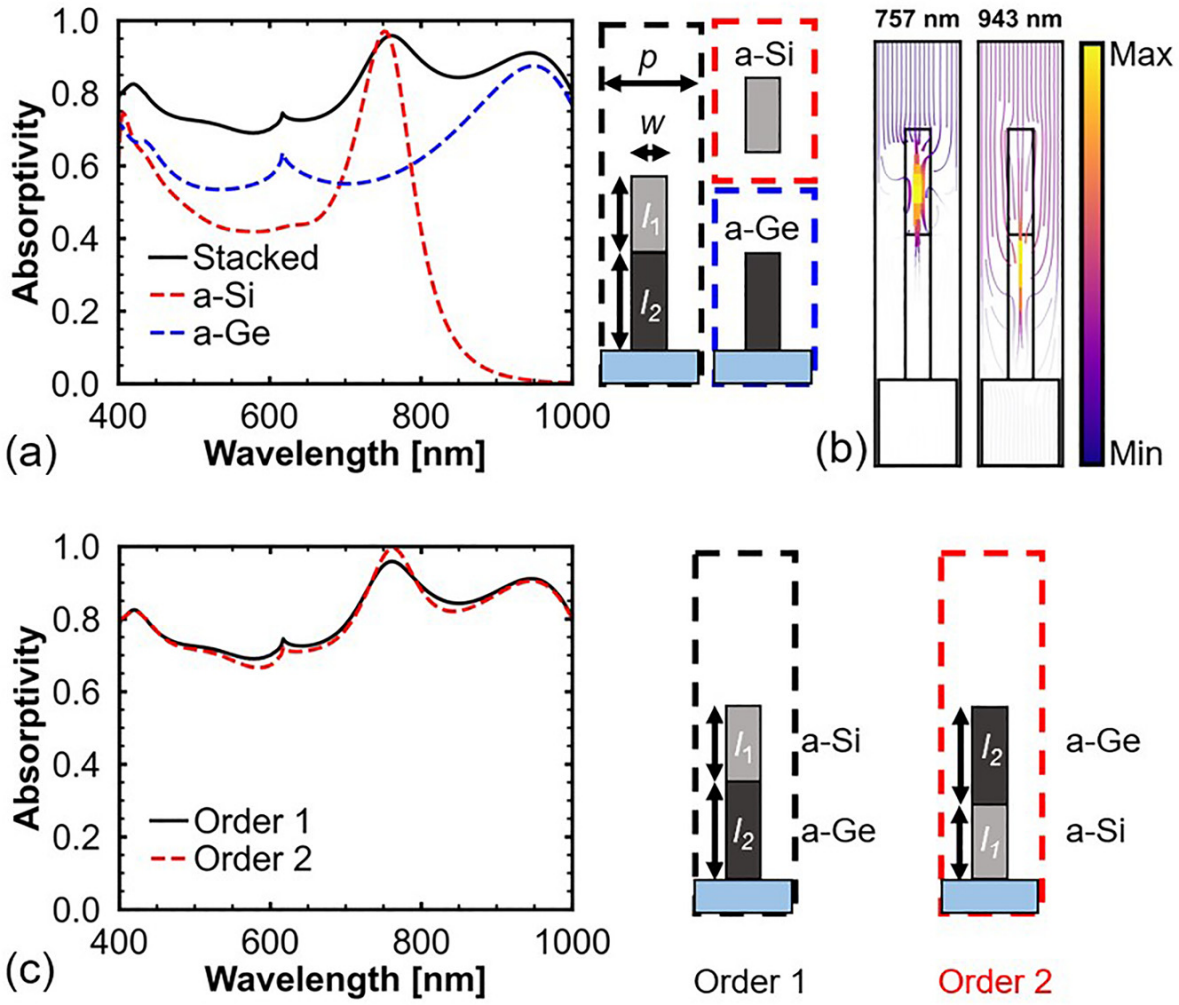
Optical properties of unstacked and stacked nanopillars. (a) Absorption spectra of unstacked and stacked nanopillars of a-Si and a-Ge. The parameters of the stacked nanopillars are *w* = 130 nm, *l*_1_ = 400 nm, *l*_2_ = 550 nm, and *p* = 400 nm. (b) Streamlines of power flow for the stacked nanopillars at 757 nm and 943 nm. (c) Absorption spectra of the stacked nanopillars with different stacking orders.

The DCC-based nanopillar perfect absorber has a characteristic degenerate critical length *L*_DC_ [30]. At *l* = *L*_DC_, the nanopillar Mie resonators achieve perfect absorption. At *l* > *L*_DC_, the peak of perfect absorption is not affected by the variation in *l*. Hence, nanopillars of different materials can be stacked to achieve a broadband absorber. Figure 5a shows the absorption spectra of unstacked and stacked nanopillars of a-Si and a-Ge (Supporting Information S5 shows the simulated absorption spectrum between 400 and 2000 nm). Because the refractive index *n* of a-Ge (*n* ∼ 4.8) [42] is greater than that of a-Si (*n* ∼ 4) [36], the absorption peaks of the two nanopillars with the same *w* are different. The absorption peak wavelengths of the unstacked nanopillars are identical to those of the stacked nanopillars. The unstacked a-Si nanopillar exhibits 97% absorption at 751 nm. Whereas, that of the unstacked a-Ge nanopillar at 943 nm is 87%, which is lower than 99%. This can be attributed to the high *k* value of a-Ge at 943 nm (*γ* < *δ*). The spectrometer in this study is capable of measuring the spectrum between 400 and 1000 nm; therefore, the a-Ge nanopillar is designed in this wavelength range. Moreover, considering the fabrication of the sample, the same *w* is chosen for both a-Si and a-Ge nanopillars. Hence, only the a-Si nanopillar achieves perfect absorption. The Fabry–Perot effect can enhance the absorption of multi-layer structures [43, 44]. However, for the stacked nanopillars, the absorption arising from the Fabry–Perot effect is negligible. This is because the nanopillars have very low reflection between 400 and 1000 nm, as shown in Figures 2d and 4b. The unabsorbed light rapidly passes through the substrate. Therefore, the broadband absorption is mainly attributed to the DCC-based perfect absorption and the attenuation during propagation.

Figure 5c shows that broadband absorption can be achieved regardless of the stacking order of the a-Si and a-Ge nanopillars. The absorptivity at 761 nm for the nanopillars with stacking order 2 is 0.99, which is slightly larger than that for stacking order 1. This is because, for stacking order 1, the a-Si nanopillar does not achieve perfect absorption, and a small fraction of the incident light is reflected. Whereas, for stacking order 2, the reflected light from the a-Si nanopillar is absorbed by the a-Ge nanopillar. However, to achieve photon sorting, the a-Si nanopillar that absorbs short-wavelength light must be placed on top of the a-Ge nanopillar that absorbs long-wavelength light. Figure 5b shows streamlines of the power flow of the stacked nanopillars at the wavelengths of the absorption peaks. At 757 nm, the incident light is almost completely absorbed by the a-Si nanopillar. At 943 nm, the incident light is not absorbed by the a-Si nanopillar because the *k* value of a-Si at this wavelength is very low. Moreover, as shown in Figures 2d and 4c, the a-Si nanopillar exhibits high transmissivity at wavelengths greater than 800 nm owing to the low filling factor. Hence, the incident light at 943 nm can be absorbed by the a-Ge nanopillar. Further, at 757 nm, the material loss of a-Ge is high. If the a-Ge nanopillar is placed on top of the a-Si nanopillar, a certain portion of the incident light is absorbed by the a-Ge nanopillar before reaching the a-Si nanopillar. Hence, there is no selective absorption at 757 nm (Supporting Information S6 presents a comparison of absorption profiles between the stacked nanopillars with different stacking orders).

**Figure 6:**
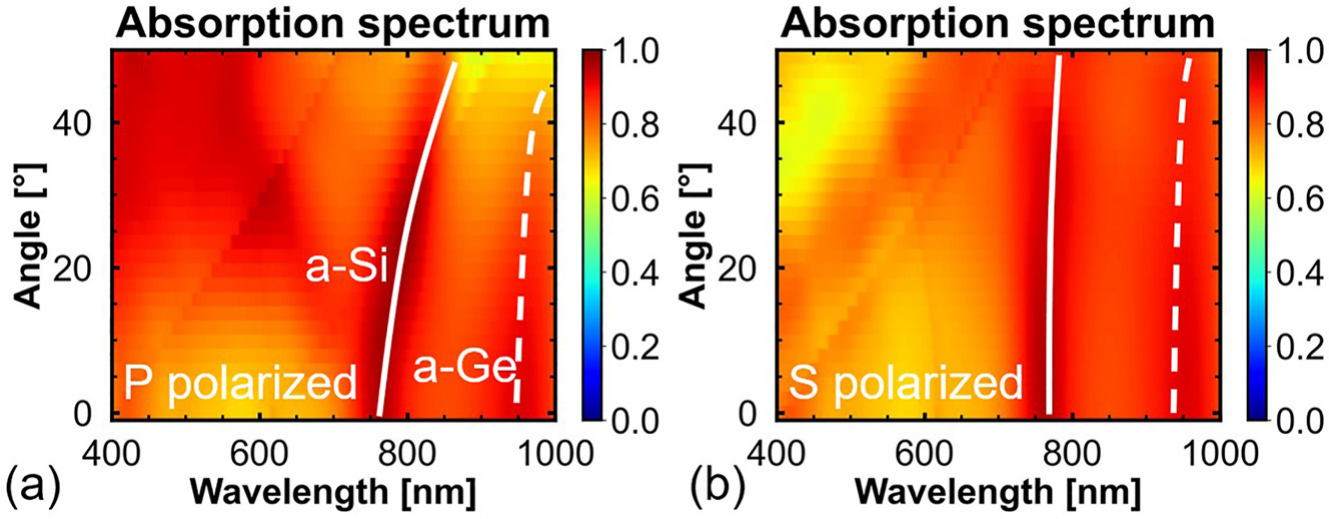
Angle-dependent absorption spectral maps of the stacked nanopillars with stacking order 1 under (a) *p*-polarized and (b) *s*-polarized light.

Figure 6 shows the angle-dependent absorption spectral maps of the stacked nanopillars with stacking order 1. As shown in Figure 6a, the absorption peaks of the a-Si and a-Ge nanopillars have a small redshift with increasing incident angles of *p*-polarized light. Figure 6b shows that the absorption of the stacked nanopillars is hardly affected by the change in the incident angle of *s*-polarized light. Hence, we can conclude that the influence of non-normal incident light (0–8.6°) introduced by the objective lens in the experiment on the final measured spectra is negligible.

**Figure 7:**
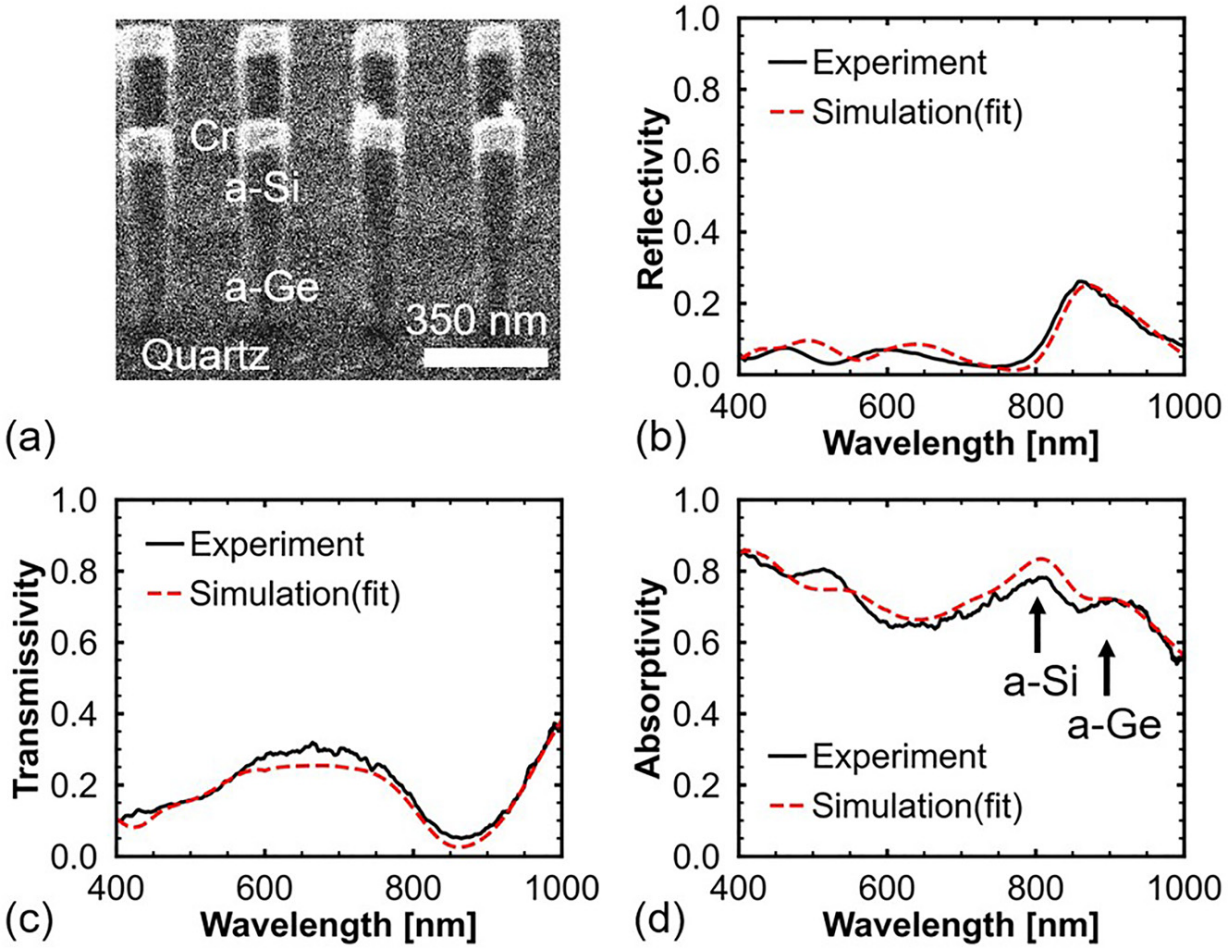
Experimental results of the proposed stacked nanopillars. (a) Focused ion beam image of the stacked absorber taken at an angle of 40°. (b) Measured reflection and (c) transmission spectra of the fabricated sample. (d) Absorption spectrum of the fabricated sample, which is calculated by 1 − *T* − *R*.

A sample was fabricated to demonstrate the performance of the proposed stacked perfect absorber. Figure 7a shows an image of the fabricated sample captured at an angle of 40° using a focused ion beam system. The goal was to fabricate a stacked absorber comprising a-Si and a-Ge nanopillars, and a quartz substrate. However, chromium (Cr) caps, which were used as a hard mask during the etching process, remain on top of the fabricated stacked absorber. This is because the etchant (Pure Etch CR 101) used to remove the Cr caps can also remove a-Ge nanopillar Mie resonators. Hence, the use of this solution for Cr etching was skipped.

Figure 7b–d show the measured reflection, transmission, and calculated absorption spectra, respectively, of the fabricated sample. Numerical simulations were performed to investigate the measured spectra. The simulated spectra are plotted as red dashed lines in Figure 7. The simulated results are consistent with the measured results. The 30 nm-thick Cr caps have a limited effect on the spectra. Thus, the Cr caps slightly increase the reflection at 850 nm, and the absorption of the stacked absorber decreases (Supporting Information S7 presents the spectra of the stacked nanopillars with and without the Cr caps).

Figure 7d shows two absorption peaks at 800 and 880 nm. The absorption peak at 800 nm is attributed to the a-Si nanopillar. However, the absorptivity of the a-Si nanopillar is not very high because the *k* of a-Si at 800 nm (*k* ∼ 0.1, *γ* > *δ*) is smaller than the *k* at 709 nm (*k* ∼ 0.23, *γ* ≈ *δ*). Further, the incident light is not completely attenuated by the a-Si nanopillar but continues to propagate forward. Hence, the a-Ge nanopillar also contributes to the absorption at 800 nm. The a-Ge nanopillar exhibits an absorption peak at 880 nm, and the absorptivity is 0.7. This is mainly because the *k* value of a-Ge at 880 nm is large (*k* ∼ 0.72, *γ* < *δ*); therefore, the conditions of the DCC are not fully satisfied. The absorption peak wavelengths of the a-Si and a-Ge nanopillars are very close compared to that of the simulated results shown in Figure 5a. This is probably attributed to the fact that the *w* of the fabricated a-Ge nanopillar is smaller than that of the a-Si nanopillar. The protection gas recipe is optimized to protect Si from lateral etching; hence, the lateral protection of a-Ge is inferior to that of Si. The smaller *w* causes the blue shift in the absorption peak of the a-Ge nanopillar.

Table 1 compares different broadband absorbers in the visible and NIR ranges with a theoretical average absorptivity of over 80%. Because energy is crucial to our society, much study has focused on broadband absorbers for use in solar cells. However, in addition to broadband absorption, the stacked nanopillars in this study are capable of photon sorting, which extends the potential applications of broadband absorbers to photodetection and imaging. In addition, it is noteworthy that the stacked nanopillars achieve broadband absorption without metal mirrors, greatly contributing to the simplification of broadband absorber structures.

**Table 1:** Comparison of broadband absorbers in the visible and NIR ranges.

	Type	Layers	Thickness [μm]	Bandwidth [nm]	Photon sorting	Metal mirror	Ref.
Metasurface	DCC (stacked)	2	0.95	400–1000	Yes	No	△
(Dielectric)	ARC	2	–	450–900	No	No	[45]
Metasurface	DMDM	4	0.325	400–800	No	Yes	[46]
(Plasmonic)	MDM (stacked)	21	0.5	480–1480	No	Yes	[47]
Nanocone	ARC	2	4.5	400–1400	No	No	[48]
Graphene	Guided modes	62	0.27	400–2500	No	Yes	[49]
Nanotube	ARC	1	∼300	457–633	No	No	[50]

DCC, degenerate critical coupling; △, this study; ARC, anti-reflection coating; DMDM, dielectric-metal-dielectric-metal; and MDM, metal-dielectric-metal.

## 3 Conclusions

In conclusion, this study proposed an a-Si perfect absorber based on the DCC comprising nanopillar Mie resonators. Nanopillar perfect absorbers have a characteristic *L*_DC_ [30]. At *l* = *L*_DC_, nanopillar perfect absorbers can achieve perfect absorption. At *l* > *L*_DC_, the absorption peak of nanopillar perfect absorbers is not affected by increasing *l*. Hence, the a-Si and a-Ge perfect absorbers can maintain their perfect absorption after stacking. Consequently, the absorption peaks of the stacked absorber become broader. The study shows that a DCC-based perfect absorber can achieve broadband absorption, which has great potential for energy applications. In addition, if the a-Si perfect absorber is placed on top of the a-Ge perfect absorber, the stacked perfect absorber can selectively absorb light of different wavelengths like a photon sorter, which has great potential for imaging applications. Thus, this study provides an additional parameter to engineer the optical properties of dielectric perfect absorbers, that is, dielectric perfect absorbers can be extended from 2D to 3D structures.

## Supplementary Material

Supplementary Material Details
