# Supplementary material for: Vertical photon sorting by stacking silicon and germanium nanopillars for broadband absorbers
Source: Nanophotonics. 2023 Mar 27;12(13):2461–9. doi: 10.1515/nanoph-2023-0014 (PMC11501955; doi:10.1515/nanoph-2023-0014)
Supplement: Supplementary file 1 — Supplementary Material Details [file j_nanoph-2023-0014_suppl_001.docx]

**Supplementary for**

**Vertical photon sorting by stacking silicon and germanium nanopillars for broadband absorbers**

Rongyang Xu^1^, Takumi Morimoto^1^, and Junichi Takahara^1, 2 *^

*^1^ Graduate School of Engineering, Osaka University, 2-1 Yamadaoka, Suita, Osaka 565-0871, Japan*

*^2^ Photonics Center, Graduate School of Engineering, Osaka University, 2-1 Yamadaoka, Suita, Osaka 565-0871, Japan*

^*^ E-mail: [takahara@ap.eng.osaka-u.ac.jp](mailto:takahara@ap.eng.osaka-u.ac.jp)

**S1. Settings of FDTD simulations**

Figure S1 shows the model of the FDTD simulation used in our study. We use a plane wave light source. The incident light is polarized in the x-direction and propagates in the z-direction. A monitor used to calculate the reflection spectrum is placed above the light source. A monitor used to calculate the transmission spectrum is placed below the Mie resonator. Absorption is calculated by 1−reflection−transmission. A mesh override region with a mesh size of 1 nm is used to improve the accuracy of the simulation. The boundary conditions (BCs) in the z-direction are perfectly matched layers (PML), which attenuate the light without reflection. The BCs in the x- and y-directions are anti-symmetric and symmetric BCs, which makes the simulation four times faster.


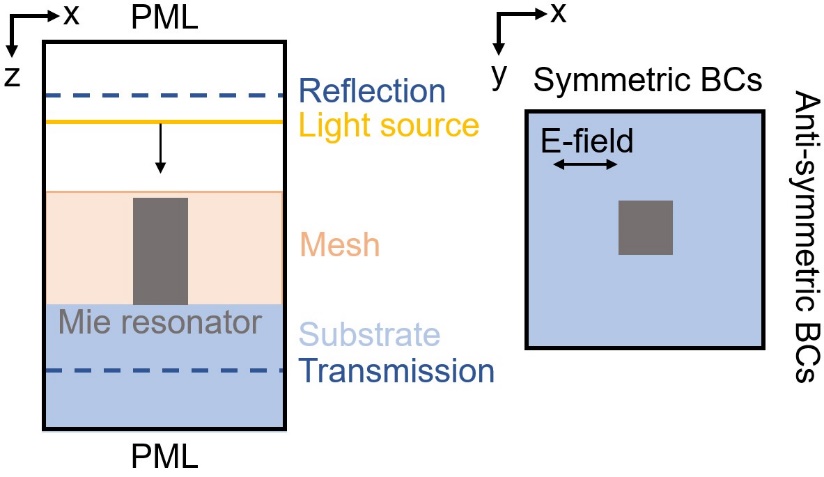


**Figure S1.** The setting of FDTD simulations.

**S2. Process of sample fabrication**

Figure S2 shows the fabrication process of the sample. We deposited amorphous silicon (a-Si) on a quartz substrate by RF sputtering (SVC-700LR). After spin-coating with a 200 nm-thick chemical resist (ZEP520A-7), the sample was baked on a hot plate at 180 °C for 2 minutes. We exposed the sample by electron beam lithography (ELS-100T, Elionix), and the resist in the exposed area was removed after 60 seconds of development in solvent (ZED-N50, Zeon). A 35 nm-thick Cr layer was deposited as a hard mask for etching on the top of the sample by electron beam deposition (UEP-2000 OT-H/C, Ulvac). After removing the resist with N-methyl-2-pyrrolidone (NMP) solvent, we etched the sample with SF_6_ and C_4_F_8_ gases in an etching chamber (ELS-700, Elionix). Finally, we removed the Cr mask with an etchant (Pure Etch CR101, Hayashi Pure Chemical).

**
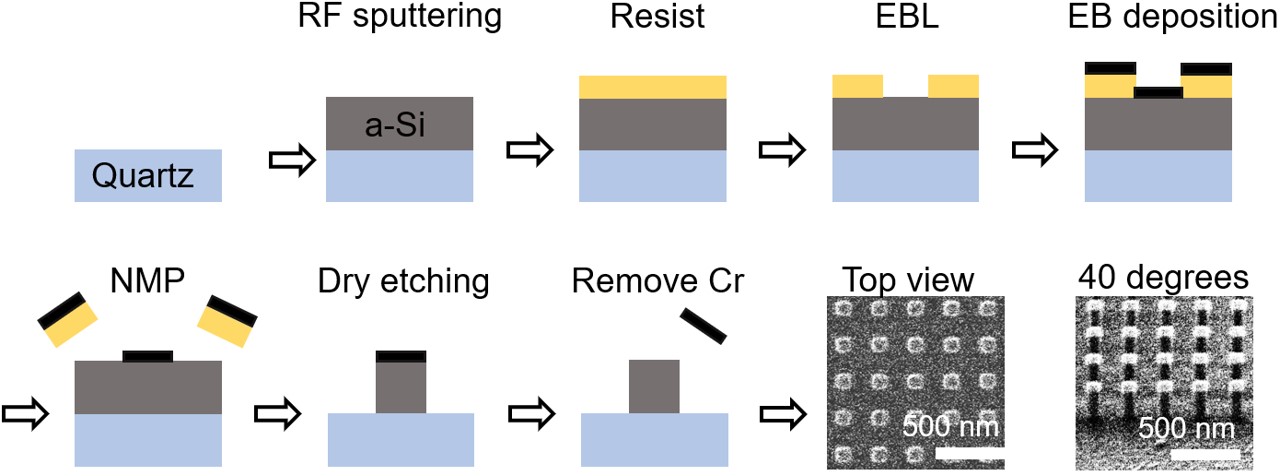
**

**Figure S2.** The process of fabricating the sample.

**S3. Measurement setup for reflection and transmission spectra**

**
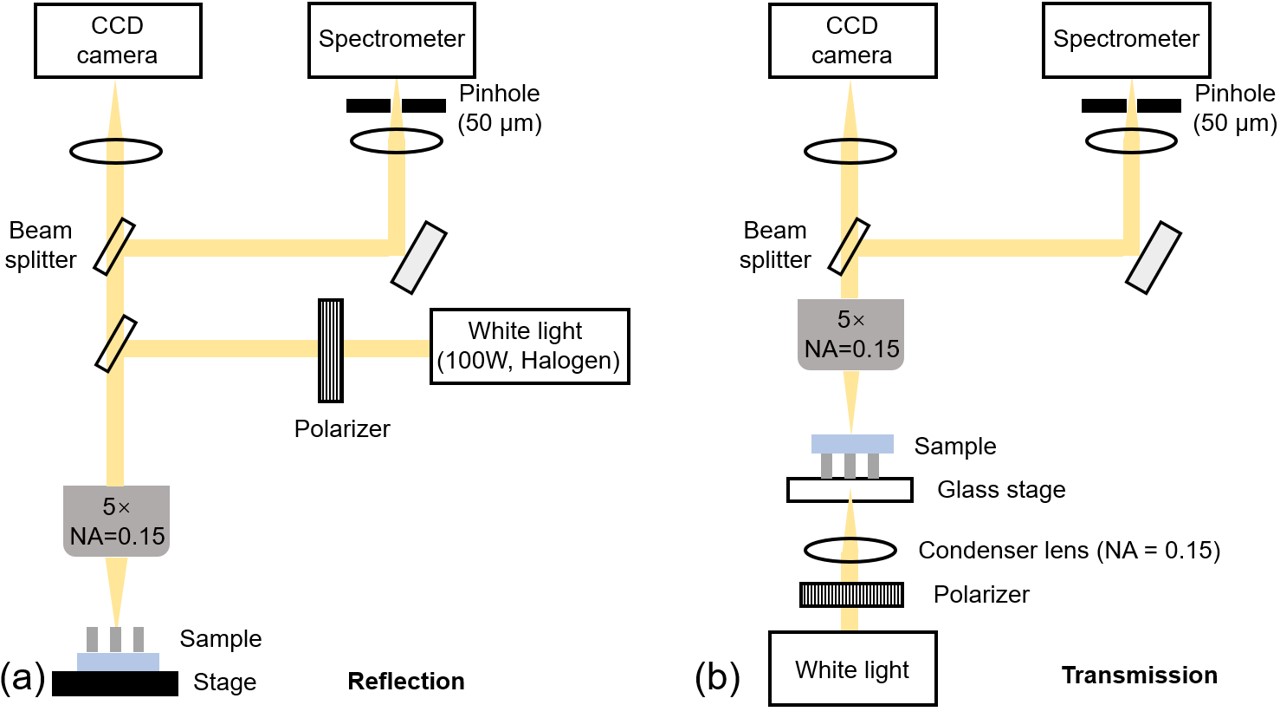
**

**Figure S3.** Schematic of the measurement setup for (a) reflection and (b) transmission spectra.

In this study, absorption is calculated by 1−reflection−transmission. Figure S3a shows the schematic of a setup for measuring reflection spectra. We used a white light source for observation and measurement, and the incident light was linearly polarized after passing through a polarizer. Then, the incident light was focused by an objective lens with numerical aperture NA = 0.15 (incident angle: 0−8.6°). The fabricated sample was placed on a black metal plate. The light reflected from the sample *R*_sample_ was collected by the same objective lens. The collected light passes through a pinhole with a diameter of 50 μm and reached a spectrometer of visible light. A protected silver mirror was used as a reference for reflection measurement *R*_reference_. We can obtain the final reflection spectrum of the sample by *R* = *R*_sample_/*R*_reference._

Figure S3b shows the schematic of a setup for transmission measurement. After passing through a polarizer, the incident light was focused by a condenser lens with NA = 0.15. The light passed through the sample *T*_sample_ and was collected by the objective lens of the same NA. Finally, the light reached the spectrometer of visible light. A quartz substrate of the same thickness as the quartz substrate of the sample was used as a reference *T*_reference_. The final transmission spectrum of the sample *T* is calculated by *T* = *T*_sample_ /*T*_reference_.

**S4. Fabrication errors of nanopillars**

Fabrication errors of the nanopillars lead to the difference between the simulated and measured results. As shown in Figure S4, the upper-end width of the fabricated nanopillars is wider than the width used in the simulations. This is due to a longer dose time used in the process of electron beam lithography (EBL), resulting in a wider width of the exposed square patterns. Hence, the width of the Cr masks fabricated by electron beam (EB) deposition is more than 120 nm. After dry etching, the upper-end width of the nanopillars is also wider than 120 nm. In addition, the sidewalls of the fabricated nanopillars are slightly tilted because our dry etching recipe was not optimized for fabricating nanopillars. The lateral etching makes the lower-end width of the nanopillars close to 120 nm. Figure S5 shows the absorption spectral map of the nanopillars with varying upper-end widths. The peak wavelength of the absorption peak increases with increasing the upper-end width. The absorptivity of the a-Si perfect absorber is 90% at the upper-end width of 140 nm. Hence, we can conclude that the fabrication errors have little effect on the absorption spectra of the nanopillars.


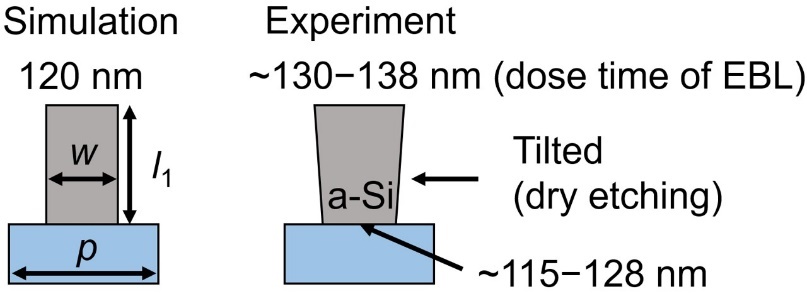


**Figure S4.** Schematic of the nanopillars in the simulations and the experiments.


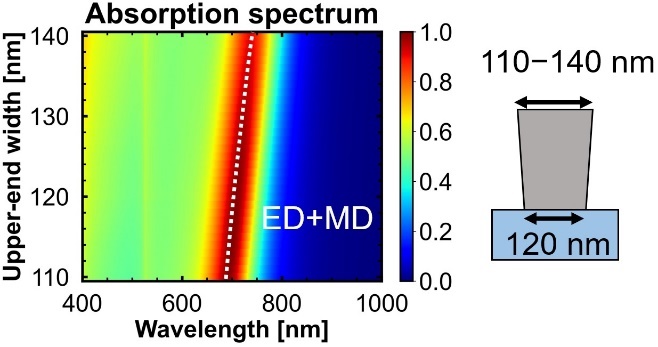


**Figure S5.** Absorption spectral map of the nanopillars with upper-end widths between 110 and 140 nm.

**S5. Absorption spectrum of stacked nanopillars**

Figure S6a shows the spectra of the stacked nanopillars in the range of 400−2000 nm. There are two reasons why the absorption of the stacked nanopillars decreases at wavelengths beyond 1000 nm. First, resonant modes can significantly enhance the light-matter interaction, but no resonant modes are excited at longer wavelengths. Second, the material loss of a-Si and a-Ge decreases rapidly with increasing wavelength in this range, as shown in Figure S6b.


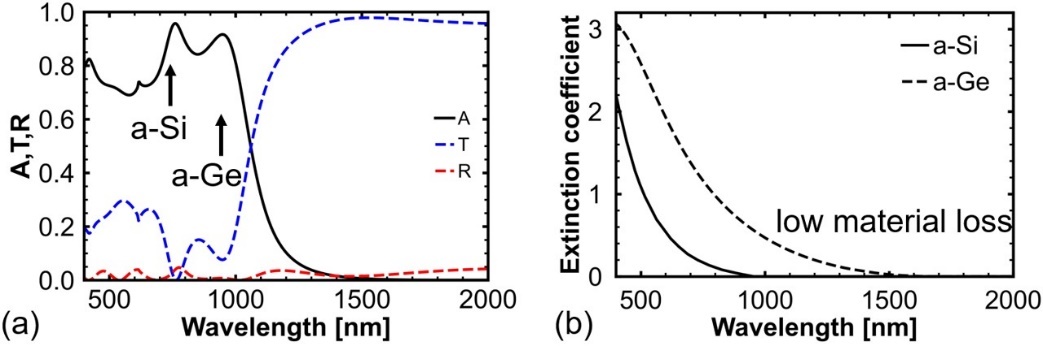


**Figure S6.** (a) Absorption, transmission, and reflection spectra of the stacked broadband absorber. (b) Extinction coefficient of a-Si [1] and a-Ge [2].

**S6. Absorption spectra and absorption profile of different stacking orders**

**
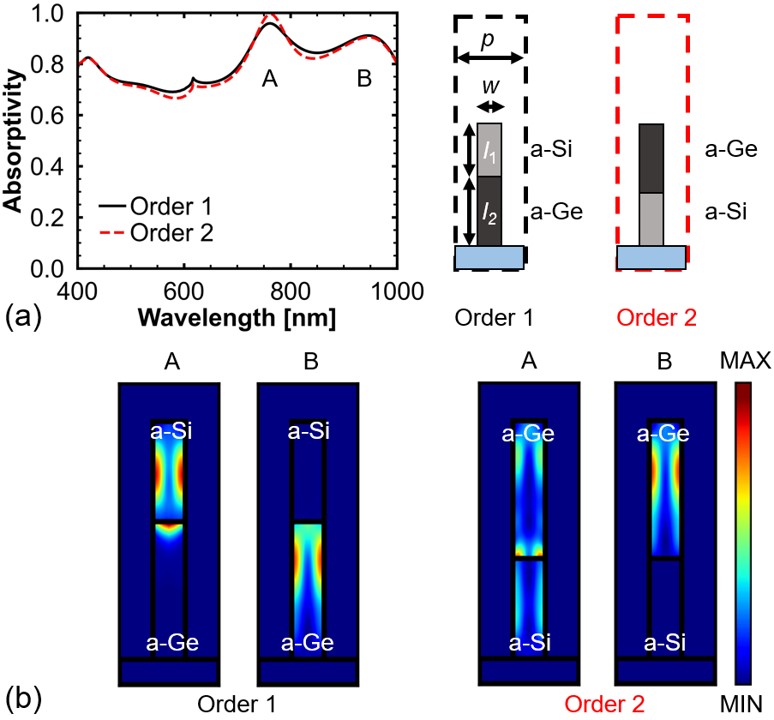
**

**Figure S7.** (a) Calculated absorption spectra with the different staking orders of the a-Si and a-Ge nanopillars. (b) Absorption profiles of the stacked nanopillars of different stacking orders.

Figure S7a shows the absorption spectra of the stacked nanopillars with different stacking orders. For the stacked nanopillars consisting of a-Si and amorphous germanium (a-Ge), the absorption spectra are almost the same for different stacking orders. This is because the reflection of the a-Si and a-Ge nanopillars is very low between 400 to 1000 nm. If the a-Ge nanopillar is placed on top of the a-Si nanopillar, at 757 nm, part of the incident light is absorbed by the a-Ge nanopillar during propagation. The rest of the light reaches the a-Si nanopillar and is completely absorbed by the a-Si nanopillar. Figure S7b shows the absorption profiles at the wavelengths of the absorption peaks for the stacked nanopillars of different stacking orders. We can conclude that the nanopillar with a shorter operating wavelength has to be placed on top of the nanopillar with a longer operating wavelength to achieve the photon sorting capability.

**S7. Effect of the Cr caps on the spectra**

**
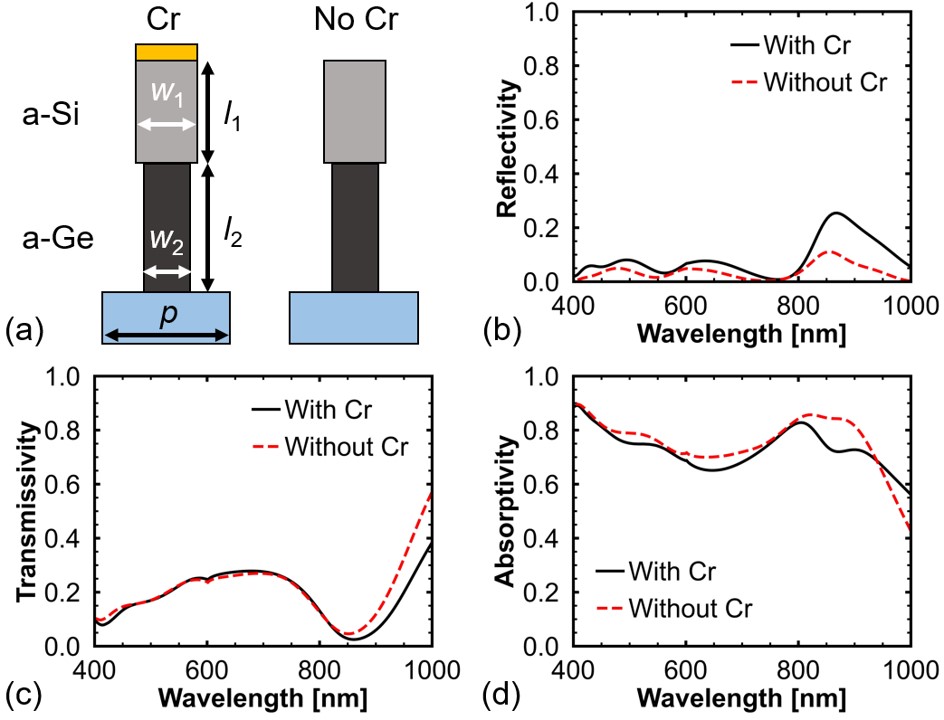
**

**Figure S8.** (a) Schematic of the stacked nanopillars with and without Cr caps. The parameters are *w*_1_ = 150 nm, *w*_2_ = 110 nm, *l*_1_ = 405 nm, *l*_2_ = 545 nm, and *p* = 390 nm. Calculated (b) reflection, (c) transmission, and (d) absorption spectra for the stacked nanopillars with and without Cr caps.

Figure S8 shows the spectra of the stacked nanopillars with and without the 30 nm-thick Cr caps. The Cr caps have little effect on the spectra of the stacked nanopillars. At 850 nm, the introduction of the Cr caps enhances the reflection and slightly reduces the absorption.

**Reference:**

[1] Pierce D T, Spicer W E, Electronic Structure of Amorphous Si from Photoemission and Optical Studies. Physical Review B 1972, *5*, 3017–3029.

[2] Ciesielski A, Skowronski L, Pacuski W, Szoplik T, Permittivity of Ge, Te and Se thin films in the 200–1500 nm spectral range. Predicting the segregation effects in silver. Materials Science in Semiconductor Processing 2018, *81*, 64–67.
